# Exploring the causes of work-related stress and burnout among doctors in Bangladesh: a qualitative study

**DOI:** 10.1080/17482631.2026.2616350

**Published:** 2026-01-14

**Authors:** Pragna Paramita Mondal, Tasnima Haque, Judith Johnson, Atiya Rahman, Kaosar Afsana, Raghav Mistry, NgaMan Chan, Olga Lainidi

**Affiliations:** a BRAC James P Grant School of Public Health, BRAC University, Dhaka, Bangladesh; b University of Copenhagen, Copenhagen, Denmark; c University of Manchester, School of Health Sciences, Manchester, UK; d University of Leeds, School of Psychology, Faculty of Medicine and Health, Leeds, UK

**Keywords:** Bangladesh, doctors, burnout, mental health awareness, qualitative

## Abstract

**Purpose:**

The global shortage of healthcare professionals disproportionately affects low/middle income countries. Bangladesh is facing critical health workforce shortages, exacerbating workload and the risk of doctors’ burnout. However, there is a lack of qualitative research into causes of occupational burnout in Bangladeshi doctors. This study investigated the factors contributing to burnout among Bangladeshi doctors.

**Methods:**

An exploratory approach was employed using Reflexive Thematic Analysis with a Critical Realist approach. Fifteen semi-structured interviews were conducted with Bangladeshi doctors (general practitioners, cardiologists, surgeons, and paediatricians). Data were collected in English or Bangla and analysed using Atlas.ti version 24.

**Results:**

Four themes were developed: (1) the postgraduate phase is a pressure pinch-point, (2) there is limited awareness of mental health issues and insufficient support, (3) high workload and competing demands, (4) unhelpful public attitudes and media narratives. Findings highlight structural, cultural, and organizational factors driving burnout.

**Discussion:**

Addressing burnout in Bangladeshi doctors requires systemic and policy-level interventions. Mental health support, workload management strategies, and public awareness initiatives are critical to improving doctors’ well-being and sustaining the healthcare workforce in Bangladesh. Overall, the study offers the first in-depth qualitative account of how intersecting structural and cultural pressures shape doctors’ experiences of burnout in Bangladesh.

## Introduction

The shortage or absence of healthcare providers is a public health challenge that is increasingly causing higher workload burden for the providers who remain. There will be an estimated global deficit of 10 million nurses, midwives and doctors by 2030, which will disproportionately affect low-and-middle-income countries (World Health Organisation, 2022). While insufficient levels of recruitment and training have been identified as important factors, high turnover is the dominant contributor to healthcare workforce shortages (Castro Lopes et al., 2017). Work stress and burnout increase the risk of turnover (Hodkinson et al., 2022; Vogt et al., 2023), with recent evidence suggesting burnt-out physicians are three times as likely to intend to leave their job (Hodkinson et al., 2022). These findings are concerning; levels of stress and burnout in healthcare professionals increased during the pandemic and have yet to return to pre-pandemic levels (General Medical Council, 2023).

The term ‘work stress’ refers to workers’ experiences when the demands they are facing outstrip the psychological, physical, social and organisational resources they have available (Demerouti & Bakker, 2011). Burnout is considered to be a syndrome which occurs when workers are under prolonged work stress (Maslach & Leiter, 2016). The two main dimensions of burnout are emotional exhaustion, a state of emotional and physical depletion, and disengagement or depersonalisation, a state of reduced interest and personal investment in work and patients (Demerouti & Bakker, 2008). In addition to impacting staffing levels, stress and burnout in providers can impact the quality and safety of patient care delivery (Hodkinson et al., 2022). A recent systematic review and meta-analysis of 170 studies found that physicians experiencing high levels of burnout were twice as likely to be involved in a patient safety incident and twice as likely to have dissatisfied patients (Hodkinson et al., 2022). Qualitative research has identified mechanisms which may underlie the association between burnout and poorer patient safety, suggesting that high burnout can decrease cognitive functioning, increasing the risk of mistakes (Hall et al., 2017a). This research also suggests that burnout can lead to negative provider attitudes towards patients, reducing the opportunity for good communication and further increasing the risk that a mistake will be made, such as a missed diagnosis (Hall et al., 2017a).

Most of the burnout research conducted to date has been in high income countries. Qualitative studies in these settings have shown that burnout often stems from high workload, administrative burden, limited work–life balance, and challenges in professional culture (Al-Ghunaim et al., 2022; Hall et al., 2017b). There has been a disproportionate lack of research on this topic within low-and-middle income countries, where the majority of the world’s population resides (Naji et al., 2021; Woo et al., 2020). While these findings from high-income countries provide valuable insights, they may not fully capture the experiences of physicians in low- and middle-income countries, where health system constraints, resource limitations, and cultural dynamics may play a greater role. For example, in a 2019 global systematic review and meta-analysis of burnout prevalence studies in postgraduate medical trainees, three-quarters of included studies were conducted in North America, Europe or Australia and New Zealand (Naji et al., 2021). Furthermore, there is a pronounced paucity of studies on this topic in countries defined as ‘least developed’ according to the Organisation for Economic Co-operation and Development (OECD) Development Assistant Countries (DAC) list of Official Development Assistance (ODA) recipients (OECD, 2024). For example, in a 2020 systematic review of 113 global studies into burnout in nurses, only one study included countries considered ‘least developed’ (Woo et al., 2020). As these are the countries most vulnerable to healthcare professional shortages globally, there is a need for greater understanding of burnout in these nations.

Bangladesh is a country with a population of 170 million, which is categorised as ‘least developed’ according to the OECD DAC list of ODA recipients (OECD, 2024), and which is experiencing a healthcare worker shortage. The World Health Organisation (WHO) estimates there are 7 doctors per 10,000 of the Bangladesh population, compared with 32 per 10,000 in the UK and 41 per 10,000 in Australia (WHO, 2023). Altogether, WHO (2021) estimates there are just 49 healthcare workers of any type per 10,000 of the Bangladesh population, including those classed as ‘non-qualified and unrecognised. The result is that Bangladesh healthcare professionals experience a high workload burden and the potential for work-related stress and burnout (Naji et al., 2021; Rashid et al., 2022). There have recently been several quantitative studies into provider burnout in Bangladesh (Rashid et al., 2022; Rizwan et al., 2023; Nobi et al., 2023; Hutchings et al., 2024). These have suggested that up to half of Bangladesh healthcare professionals are at risk of burnout, with female physicians, early career or younger professionals, those working longer hours or with higher workloads and those experiencing job dissatisfaction at the highest risk. However, there remains no qualitative investigation into the causes and experiences of burnout among Bangladeshi doctors. Addressing this gap is essential, as qualitative approaches can capture the cultural and systemic factors driving burnout that may not be reflected in quantitative survey tools developed in other contexts.

The World Bank Data suggest that the Bangladeshi healthcare workforce is predominantly physician-centric, with approximately 5.3 physicians per 10,000, and this seems to have had an upward trend especially in the last 20 years (World Bank, n.d.). Bangladesh's healthcare system is currently undergoing significant reforms, with the Ministry of Health and Family Welfare (MOHFW) and the World Health Organisation (WHO) collaborating to develop the nation's first National Medical Education Strategy (United Nations Bangladesh, 2024). Therefore, studying job stress and burnout among Bangladeshi doctors is essential to inform targeted interventions that can enhance physician well-being, improve healthcare delivery, and support the successful implementation of the National Medical Education Strategy. Thus, the current study aimed to explore the causes leading to doctors’ work-related stress and burnout in Bangladesh.

## Materials & methods

### Design

A qualitative exploratory design was used. Qualitative research was chosen because it enables in-depth exploration of lived experiences and contextual influences. This is particularly important given that widely used burnout measurement tools were originally developed in Western settings and have not yet been validated in Bangladesh (Demerouti & Bakker, 2008; Maslach & Jackson, 1981; Schaufeli et al., 2020). Furthermore, while intervention studies have demonstrated effectiveness in high-income countries, cultural adaptations are likely to be required to address the needs of Bangladeshi physicians (Johnson et al., 2023; Panagioti et al., 2017; Al-Ghunaim et al., 2023; Vogt et al., 2024). Qualitative methods therefore, provide a suitable approach to reflect cultural nuances and inform context-specific interventions (Copeland et al., 2021).

### Participants

This study involved a diverse group of Bangladeshi doctors to gain a broad understanding of their experiences with work-related stress and burnout. The study was conducted in a range of settings, including private and government hospitals, and doctors' private practices. Furthermore, some interviews were conducted via Zoom with doctors based outside Dhaka, to enable access to a wider geographical group of participants.

Participants were selected using purposive sampling to ensure a diverse sample (Etikan et al., 2016; Martinez-Mesa et al., 2016). Remote interviewing enabled participation from doctors working in geographically dispersed or hard-to-reach areas of Bangladesh, including smaller towns and rural hospitals, which would have been logistically difficult to access in person.

A maximum variation (or maximum heterogeneity) approach was adopted within the specific population of Bangladeshi doctors. This strategy aimed to capture a broad range of perspectives across professional roles (e.g., general practitioners, cardiologists, surgeons, paediatricians), career stages, and institutional contexts (public and private sectors). Such diversity within a defined population is well-established as an appropriate and rigorous strategy in Reflexive Thematic Analysis (RTA), as it facilitates the identification of shared patterns of meaning that transcend surface-level demographic or contextual differences (Braun & Clarke, 2022). Participants included general practitioners, cardiologists, surgeons, and paediatricians with varying years of experience, working in both public and private hospitals. To capture a broader range of perspectives, both junior and senior doctors were interviewed. In this study, junior doctors were defined as those with up to 12 years of experience working as a doctor, primarily as general practitioners (GPs), postgraduate trainees, or individuals who had recently completed their postgraduate degrees. Doctors with more than 12 years of experience working as a doctor were classified as senior doctors.

Sample adequacy was guided by the principle of information power (Malterud et al., 2016), which emphasises the appropriateness of both the sample composition and size in qualitative research. According to this principle, the more information power the sample provides, the smaller the sample size required. Unlike information redundancy or data saturation, which are rooted in post-positivist assumptions of discovering a fixed set of meanings, information power aligns with the interpretive and constructivist assumptions underpinning Reflexive Thematic Analysis (RTA) (Braun & Clarke, 2022). It focuses on the richness, relevance, and quality of the data in relation to the study’s aims. Specifically, the study’s narrow focus on burnout among doctors in Bangladesh (rather than all healthcare workers or general mental health) enhanced the relevance of the data. The use of a Critical Realist epistemology and RTA further prioritised interpretive depth over breadth, reducing the need for a large sample. We applied the information power framework iteratively during data collection. We initially aimed to recruit between 12 and 20 participants, consistent with the study’s focused scope. After 15 interviews, the research team reviewed the dataset in relation to Malterud et al. (2016) five information power dimensions: study aim, sample specificity, use of theory, quality of dialogue, and analytic strategy. At this stage, interviews were judged to provide sufficient depth, variation, and conceptual richness to address the research question. The team therefore concluded that adequate information power had been achieved, and recruitment ended at *n* = 15.

The study included a total of fifteen participants (Table 1), with 9 from government hospitals and 6 from private hospitals. Among them, eight were junior doctors (three female and five male), and seven were senior (four female, three male). Most participants' ages ranged from 30 to over 60 years, with the majority between 31 and 45 years and one participant over the age of 60 years. Twelve participants were from Dhaka, three were from Brahmanbaria, Sylhet and Barisal, and the mean years of professional experience was 15.

**Table 1.** Demographic profile of the study participants.

Name	Age	Gender	Specialty	Years of experience
Participant 1	31–35	Female	General Practitioner	6
Participant 2	31–35	Male	Internal Medicine	8
Participant 3	60+	Female	Obstetrics and Gynaecology	40
Participant 4	31–35	Female	clinical embryology	4
Participant 5	41–45	Male	Nephrology	19
Participant 6	31–35	Male	Internal Medicine	10
Participant 7	46–50	Male	Head and Neck Surgery	22
Participant 8	41–45	Female	Paediatrician	20
Participant 9	41–45	Female	Obstetrics and Gynaecology	20
Participant 10	41–45	Female	General Practitioner	19
Participant 11	31–35	Female	General Practitioner	11
Participant 12	26–30	Male	Postgraduate trainee in Medicine	5
Participant 13	41–45	Male	Cardiac surgery	20
Participant 14	36–40	Male	Orthopaedic surgery	12
Participant 15	36–41	Male	Plastic surgery	12

This study received ethical approval from the School of Psychology Research Ethics Committee at the University of Leeds (Ethics Ref No: PSCETHS-1002 accepted on 01/03/2024) and from the Institutional Review Board of the Bangladesh Rehabilitation Assistance Committee (BRAC), James *P* Grant School of Public Health, BRAC University (Ethics Ref No: IRB-2024-IS-05 accepted on 10/03/2024). Written informed consent was obtained from all participants. After explaining the study’s purpose, voluntary participation, and the right to withdraw from the study at any time during the interview, recorded verbal consent was obtained from all of the participants. As a token of appreciation for their time and contribution, small gift vouchers were provided to the participants.

### Data collection

An interview guide with open-ended questions and prompts was developed by the research team to ensure consistency and comprehensiveness across interviews (See Supplemental Material 1). This guide included questions on workload, working environment, relationships with colleagues and patients, coping strategies, and available institutional support. The guide allowed participants to freely describe their experiences, while enabling the researchers to probe further where necessary. Basic demographic information such as gender and age was also collected. A team of three researchers—a medical doctor, a psychologist, and an anthropologist—conducted all interviews together. Interviews were conducted in either English or Bangla, depending on the participant's preference. Prospective participants were contacted via phone, provided with study information, and, upon agreeing, interviewed at their preferred place and time in a private setting to ensure comfort and confidentiality. For face-to-face interviews, audio recordings were taken, and for interviews conducted via Zoom, both audio and video recordings were made. All recordings were securely stored, transcribed, and anonymised before analysis. Interviews lasted an average of 36 minutes ranging from 15 to 60 minutes. Before the interviews, recorded verbal consent was taken. A total of 15 interviews were conducted. Data collection ended when the research team agreed that sufficient information had been gathered to achieve the study's objectives, consistent with the principle of information power.

### Reflexivity statement

Credibility and trustworthiness were established through reflexivity, transparency, and analytic depth, consistent with the principles of Reflexive Thematic Analysis (RTA) (Braun & Clarke, 2021, 2022). Reflexive practices were prioritised to ensure rigour and transparency.

Our team brought different disciplinary backgrounds and included medical doctors, psychologists, and an anthropologist, ensuring a wide range of perspectives were incorporated into both our approach to the study and our analysis of the data. Our team also included members from Bangladesh (PPM, TH, AR, KA), the United Kingdom (UK) (JJ, RM, NC), and Greece (OL), so our analytic process was supported by the views of people who were both ‘insiders’ and ‘outsiders’ to the data. The data collection team included a Bangladeshi medical doctor (PPM), a Bangladeshi anthropologist (TH) and a UK psychologist (JJ). The researchers’ positionalities influenced both data collection and analysis. For example, the first author, a Bangladeshi public health researcher, shared cultural and professional understandings with participants, which helped to establish rapport and elicit candid discussions about sensitive issues such as workload pressures and public expectations of doctors. However, this shared background also required continuous reflexive awareness to avoid assuming common interpretations. The authors who are based in the UK and are trained in psychology and health systems research, brought complementary outsider perspectives that introduced theoretical distance and encouraged critical reflection on structural and cultural explanations. Regular reflexive discussions among team members helped surface these positional influences and ensured that interpretations reflected both contextual understanding and analytic rigour.

Throughout data collection and analysis, the research team maintained reflexive notes after each interview related to assumptions, positionality, and analytic decisions (e.g., the participant’s tone, context, and potential influence of power dynamics or social desirability on the data). These reflections were discussed among team members with different cultural and occupational backgrounds (e.g., PPM, TH, OL & JJ) in subsequent team meetings during the analysis, to ensure sensitivity to context and to challenge any taken-for-granted interpretations. A detailed audit trail was maintained in Atlas.ti to document coding iterations and theme development.

### Analysis

Reflexive Thematic Analysis (RTA) was used, as outlined by Braun & Clarke (2019), Braun et al. (2023), within a Critical Realist framework (Fryer, 2022), to capture both the structural realities shaping Bangladeshi doctors’ experiences of work stress and burnout and the ways in which individuals interpret and navigate these realities. An inductive approach was used to develop themes based on the subjective experiences and understanding commonalities and variations in how people perceive or describe work stress and burnout. Reflexive Thematic Analysis (RTA) provides the analytic structure whereby themes are not “discovered” in the data but constructed through researcher reflexivity, interpretation, and engagement with participants' narratives (Braun et al., 2023). While RTA organises themes based on patterns of meaning, employing the Critical Realist perspective enabled the researchers to focus on how both structural constraints and individual agency were reflected in participants’ accounts, allowing to view external factors (e.g., workload, shortage of staff) through the lens of contingency causality structures with the potential to trigger events (e.g., work stress, emotional exhaustion) (Fryer, 2022). These perspectives emphasise that stress is subjective, and different doctors may interpret and eventually response to similar workplace situations or challenges differently, influenced by their own backgrounds, professional role, and societal and cultural norms.

The data were coded and analysed using Atlas.ti version 24. Preliminary analysis began during data collection, with team members discussing their reflections on the interviews at the end of each day. Verbatim transcription of the interviews was completed, and for those conducted in Bangla, direct translation into English was performed. Three of the co-authors independently reviewed the same two transcripts to create an initial codebook using both inductive and deductive coding methods. This process also ensured intra-coder agreement, based on which the codebook was revised. At the next stage, PPM, TH & OL independently coded 4 interviews each. After coding, one team member (JJ) reviewed all coded transcripts. Matrices were developed, and initial themes were generated. Themes were reviewed and refined until agreement was reached. The team collaboratively identified and named the final themes. Investigator triangulation was applied throughout: all three researchers regularly met to compare coding decisions, discuss assumptions, and refine emerging themes. Researcher reflexivity was emphasised to ensure interpretations were co-constructed rather than assumed to be “discovered.” Quotes from the interviews were included to amplify the voices of the doctor community and support the interpretation and explanation of the narratives. Investigator triangulation allowed us to cross-check interpretations and challenge each other’s assumptions; we did not carry out member-checking with participants or triangulate our findings with other forms of data, such as observations or documents.

## Results

### Theme 1: The postgraduate phase is a pressure pinch-point

A postgraduate degree is crucial for Bangladeshi doctors after MBBS to specialise, enhance expertise, secure better job opportunities, and high salaries in both government and private sectors. However, many newly graduate doctor face significant financial struggles during this period due to limited earning opportunities.

Participants described how the academic pressure and stress of postgraduate training could be significantly exacerbated by financial strain. According to the participants, postgraduate trainees did not receive any monetary compensation; however, a recent provision offers doctors 20,000 BDT per month (approximately 167 USD) during this period. Despite this improvement, participants highlighted how the combination of an intense workload and insufficient financial support could drive individuals to consider leaving their medical careers:

*“..........I am in 4 years of my training (post-graduate), and we have total tuition of 5 years. And during my first 6 or 7 months. so much stress and I have decided that I would. (…) exit from my doctor provision. So that, there was so much work and so much less paid. And so, after 6 months, I come to counselling with my family members, my parents, and they say that you have to live with that. So. again, I continued.” (P12, male, private, junior)*

The inadequate income during this critical phase, particularly for junior doctors after graduating from medical school, impacted their well-being and contributed to significant challenges in fulfilling their families’ basic needs. Some participants described how this became even more difficult when societal and familial expectations, including marriage and starting a family, occurred. The combination of family responsibilities and lack of income could leave doctors in desperate situations:

*“…When we graduate from medical school, one is already 25-26 years old. For personal reasons or other family reasons, that boy or girl gets married. After that, when they enter the course (post-graduation), they have no income. Moreover, if they have a baby in between, sometimes the situation gets so bad that they do not have any money to buy formula milk for their children….” (P2, male, govt, junior)*

Post-graduation trainees often juggled multiple responsibilities simultaneously. While senior doctors were present in hospitals, participants said that they were the primary caregivers, especially in government facilities, where they typically managed both clinical duties and academic commitments. They described that these extended working hours contributed significantly to burnout. As one participant stated:

*“When I was a resident doctor, my day started early at 7 am with classes, followed by a morning session at 8 am. Then, I had rounds, OT duties, and admissions, which usually wrapped up around 3 pm. After a short break, I had to return for the evening session. On nights when I had duty, I covered 12-hour shifts and then followed the same morning routine. This rigorous schedule took a significant toll on my health, as I often worked 14 hours or more each day during my residency period. That time, I had no break or rest, which I have now.” (P15, male, govt, junior)*

Participants also reported that immense academic pressure and stress that came along in this post-graduation period could constitute a significant obstacle to the effective completion of the programme. Because of these overwhelming stresses, one participant said that doctors sometimes withdrew from this medical training:

*“One of my friends dropped out of the class (post-graduation) during the resident period. He was very excited and bought all the books needed to pass the period, however, he dropped out only after 3 months because the stress level was too much for him to bear. This is very common in this sector.” (P15, male, govt, junior)*

### Theme 2: Limited awareness of mental health issues and insufficient support

Participants described how Bangladeshi medical culture often discouraged open acknowledgement of stress and burnout, pushing individuals to cope privately on their own rather than seeking support. Sometimes they felt they had to counteract negative views of stress and burnout by further increasing their workload to demonstrate their competence and capability. The stigma around expressing vulnerability could lead to a vicious cycle of overwork, further exacerbating stress and burnout. One participant described how doctors experiencing burnout or struggling to keep pace with their peers often resorted to internalising their challenges and redoubling their efforts:

*“If someone is getting burned out, struggling to keep up with another 10 doctors, if someone feels like this, then he/she will try to overcome it on their own by working hard, gaining knowledge, or by doing more and more hard work. But they will not show the world that they are stressed or burned out.” (P2, male, govt, junior)*

Some participants who were junior doctors mentioned that they avoided opening up about stress and burnout as they did not see such openness modelled by their senior colleagues. Instead, they said their senior colleagues considered these pressures simply “part of the job”; those who were struggling were considered weak. This led these junior doctors to fear seeking professional help:

*“Nobody talks about it. Our senior doctors are like, oh, we did it. You have to do it too. And, like our colleagues, they're like, oh, you're weak. (…) You cannot deal with that. Yes. I am weak. I cannot deal with it. Oh, yeah I need help. And, but, no one talks about it because, everyone else will laugh” (P1, female, private, junior)*

This perspective was also echoed by senior doctor participants, who acknowledged in themselves a reluctance to recognise stress, burnout and overwork:

*“… I'm used to be in operation theatres whole night. I never said that I am tired.… I know my body cannot accommodate so much work now, but I cannot say that even never say that I'm tired. This is my duty.my colleague may say, I’m tired, whole night, I am awake doing operations (…)I feel my body will not work so much, but, mentally, I cannot I have never said I'm tired of patients…, it's not about the patient. …. I think 80% feel tired. 20% will not say that I am tired.” (P3, female, private, senior)*

Participants described how the lack of peer support exacerbated the stress and burnout of the doctors in their workplace. Some participants reported that they felt isolated in their struggles, as they sensed there was no sincere friendship among their workplace colleagues. Rivalry and mistrust were commonly experienced by participants in the workplace, who worried that discussing personal or professional difficulties could result in miscommunications or even conflict. Because of the atmosphere of continuous wariness created by this lack of trust, some participants found it challenging to receive the emotional support and companionship needed to handle their work-related stress. One participant said:

*“…Getting a colleague who's also a friend is very rare. (…) and most of the time, colleagues are always behind each other (…) that’s another thing we have to deal with because we cannot just share one thing with someone (…)that thing will be converted to something else, and you will be in trouble…So just cannot trust, colleagues at all.…. it's like always dealing with those reality shows like that.” (P1, female, private, junior)*

Furthermore, some participants described that accessing good professional support in Bangladesh was challenging. They felt that some therapists could be judgmental; it was also expensive. They felt there were no adequate resources available to help the doctors who were experiencing stress and burnout. As a result, they said that doctors were suffering on their own and did not know where to turn:

*“Also, it's hard to find good support. Some therapists can be judgmental, and there aren't adequate helpful resources available specifically for healthcare workers. So, people suffer in silence because they're afraid of being judged and don't know where to turn…. another reason is we do not have proper, help. Like, where do we go?” (P1, female, private, junior)*

### Theme 3: high workload and competing demands

Participants reported that they had to work in settings with limited resources, with a very high number of patients to care for. Therefore, they had to work long hours to handle the high patient demand with little support, which they found very difficult to manage. Overburdened healthcare systems, coupled with inadequate staffing and resource constraints, were described as leading to unsustainable working conditions which contributed to work-related stress and burnout. One participant stated:

*“We had to stick to our work, say, starts at 8 am, sharp at 8 am, we could end at 8 o'clock in the night, and 9 at night. And even if our patient needs more time, we have to stay back in the hospital. There is no limit to working hours; it almost always crosses 70 hours a week.” (P13, male, private, senior)*

As described in Theme 1, the salary structure for Bangladeshi doctors was not sufficient to fulfil their personal and social needs. While this was most felt by junior doctors, low wages and concomitant economic pressure necessitated doctors at all levels to engage in multiple jobs at once. Some participants based in government hospitals described holding additional jobs in the private sector; some participants in the private sector similarly reported holding multiple posts. As a result, there were no time left for taking rest. In general, participants described working extended working hours, which in some cases did not leave enough time between shifts for adequate sleep:

*“…After my government job… then my private practice. It is practiced in our country. in our country, it is regular practice. So when I'm working 18 by 24, 18 by 7, it is very much exhausting.” (P7, male, govt, senior)*

For junior doctors observing this, it could engender a sense of hopelessness, as they could see no real exit from the current stressors they were experiencing.

*“ I've seen senior doctors as well, I've seen my colleagues…they do not have a work life balance at all, and they do not even understand that they do not have a work life [balance]….But the problem is, with that free time, they do another job…that is private chamber practice (…) doctors are doing that…because we are underpaid.(…) The payment I get is not enough at all.(…) So that's why it's a reason. And this is how, a younger doctor, loses the work life balance.” (P1, female, private, junior)*

Beyond the pressures of their workload, participants also described coping with adverse patient outcomes, including patient deaths. Although being dedicated to their patients' well-being and wanting positive results, negative outcomes sometimes occurred despite their best efforts. One participant recounted a case of an 11-year-old girl with bone cancer who died tragically after one of her cancerous growths ruptured. Doctors in high-casualty fields such as orthopaedics and burn units were particularly susceptible to witnessing such adverse encounters. These events placed significant stress on the doctors’ shoulders:

*“…we are dealing with a patient who came. about 14 days later with a burn and about 22% burn, five years old child. We are fighting for him to bring up, we have to fight, we step down the child from ICU to HDU (high dependency unit). And from there, he again became sick. We shipped him to ICU, we again fight for him and again shipped to HDU but he did not manage to survive, and the last day, the child died.” (p14, male, govt, junior)*

Participants reported that during emergencies such as epidemics of dengue fever, both government and private hospitals were overwhelmed by the number of patients. These increases placed further strain on already overloaded health systems. Severe overcrowding could result during these times, and new wards were sometimes opened to help reduce this strain. As healthcare workers, including doctors, could also be affected by these epidemics, these surges in patient numbers often coincided with staffing shortages. Participants described being forced to take more duty hours and duties outside of their regular obligations. As a result, participants found these periods incredibly stressful:

*“…there was dengue outbreak (…) several of my colleagues fell sick (…) when someone recovered and joined, someone else felt sick. So that's why we were always short staffed, and the hospital was always, full. Yeah…hospital management had to open new floors.there were so many patients crammed in a small place…we had more floors, more beds, and more patients, but same staff… Everyone was burned out…I was so stressed and because anything could go wrong, I had to even, take files and carry those files myself because, those, ward boys were not enough in numbers…we had really narrow, time for, interventions because those emergencies were very rapid…” (P1, female, private, junior)*

Some participants reported that excessive workloads were further compounded by expectations that they would take on additional responsibilities which were outside of their core clinical responsibilities, such as performing postmortem cases. The added strain of taking on duties outside their primary role intensified their stress and increased their risk of burnout. The combination of overwhelming patient loads and additional medico-legal obligations further exacerbated these challenges, particularly in resource-limited settings. As one participant noted:

*“Yeah, for workload, I felt burnt out. In Bangladesh, in the District Sadar Hospitals, there is so much workload. As well as doctors have to deal with post-mortem cases and physical assault cases. But the number of doctors available for these responsibilities is very low.” (P2, male, govt, junior)*

### Theme 4: unhelpful public attitudes and media narratives

Some participants described experiencing pressure imposed by the media. This additional burden, combined with other sources of stress, sometimes hindered their ability to perform their duties effectively.

*“… nowadays, another burning stress in our country, the social stress(…) I had to face the media or face the public or face the administration (…) And these are also our now stressing point… pure fatigue… post operative. So, when all this distresses, picking me, then it is, actually… it is, I want to think that, it is very difficult for a surgeon to concentrate only the surgery for the patient by considering all these things.” (P7, male, govt, senior)*

Participants described how public and media narratives tended to blame doctors for problems within hospitals, ignoring the significant systemic issues which contributed to any issues patients experienced. These negative attitudes towards doctors further exacerbated their experiences of stress and burnout:

*“The general public and media tend to side with the patients, offering little support to doctors. They generalise by saying that doctors are not providing services when they should, while in reality, we lack the necessary facilities to accommodate them (…) the media nowadays publishes and broadcasts news without thorough investigation. (…) They obtain statements from patients, who usually claim to be victims, and air this perspective in the news and on social media. Journalists need to understand how the healthcare system works and how doctors served the patients.” (P9, female, govt, senior)*

Furthermore, one participant described how journalists, lacking adequate medical knowledge, sometimes fabricated stories designed to grab attention in mass media and social media. Additionally, negative attitudes towards doctors were described by some participants as occasionally leading to violence. These acts of violence tended to occur when patients had a negative outcome, and the family then blamed the doctor and felt justified to inflict harm upon them. The participant reported that these news stories fuelled anger and mistrust against the doctors, further exacerbating their stress and burnout:

*“I had a patient who was pregnant.she began experiencing labour pain at home …trying for about nine hours…she came to me the next day and was admitted as an acute emergency case. (…) I had to make a quick decision to perform the surgery because the baby’s heartbeat was no longer detectable. (…) The baby was stillborn, but that wasn't the primary issue. Due to the prolonged labour at home, the baby's body was swollen in some parts, which is common when a foetus dies in the womb. (…) However, the media published a story claiming that I, the doctor, was responsible for the swelling in the scrotum of the deceased male baby, it could easily tarnish my reputation as a doctor”(P9 female, govt, senior)*

This was less common in private hospitals but occurred in all settings. Participants found this particularly stressful as they knew they did not have complete control over patient outcomes; all care is delivered in teams. As such, they felt powerless to ensure good patient outcomes and protect themselves from harm.

*“Because of the teamwork, if any part of my team, even couldn’t handle the scenario in a proper time, in a proper way, this.I mean, these sort of things [violence against doctors] might happen, and things might get out of hand, you never know, so we have to be more cautious, and this might cause a little bit of stress on us. But it’s a part of the game, you know…” (P13, male, private, senior)*

Conversely, a doctor working at a specialised government hospital shared that he had grown accustomed to dealing with media scrutiny, compared to other participants. Although most participants described media coverage as stressful and damaging, this junior doctor noted that over time he had become accustomed to such scrutiny, suggesting variation in how these pressures are perceived. This could also be interpreted as an adaptive response associated with detachment or cynicism:

*“Initially, it was stressful, but no, now we are getting used to deal with the media and there is no stress…because we are dealing with media past six years…In our institution, it’s very common…” (p15, male, govt, junior)*

## Discussion

This study explored the causes leading to doctors’ work-related stress and burnout in Bangladesh, revealing the complex interplay of structural, cultural, and social factors that shape their experiences. Using Reflexive Thematic Analysis (RTA) within a Critical Realist approach, we developed four themes based on the doctors’ narratives, including (1) the postgraduate phase is a pressure pinch-point, (2) there is limited awareness of mental health issues and insufficient support, (3) high workload and competing demands, and (4) unhelpful public attitudes and media narratives. The findings underscore the need for structural and cultural changes to mitigate burnout among Bangladeshi doctors.

Our first theme highlighted how Bangladeshi doctors enter their careers already experiencing burnout, stress and anxiety due to the pressures during the postgraduate phase. This aligns with existing literature indicating that medical education and early career stages, particularly during specialised training, are critical periods where doctors are susceptible to heightened stress and burnout (West et al., 2016; Dyrbye & Shanafelt, 2016). It is compounded by the view that adversity during medical education is “character building” and should be endured in silence (Montgomery & Lainidi, 2022; Parekh et al., 2021). This finding is particularly concerning in the context of Bangladesh’s already strained healthcare system, where workforce shortages mean that young doctors are quickly thrust into high-pressure roles with little institutional support, reinforcing a cycle of chronic stress and attrition. While this trend is observed globally, our study adds to the literature by highlighting how this phenomenon may be particularly pronounced in Bangladesh due to the combination of financial instability and systemic workforce challenges. Specifically, the lack of pay during this phase - though recently addressed with some financial support after enduring a series of protests by the post-graduate trainee doctors (Dhaka Tribune, 2025)—was identified by the participants as a key driver of burnout, suggesting that better financial remuneration is needed to alleviate stress in this critical period. This is in line with a Kenyan study indicating that financial pressures, in addition to long working hours and clinical expectations, contribute significantly to burnout (Muteshi et al., 2024). It is also in line with evidence from other low- and middle-income countries (LMICs) or Asian healthcare systems as well; for example, in Pakistan, medical students are 2.5 times more likely to report burnout symptoms compared to their nursing counterparts (Mufarrih et al., 2024) and medical students in China face disproportionately high mental health burdens due to extended study years and clinical demands (Zhang et al., 2024).

The second theme revealed the lack of both mental health awareness and mental health support for Bangladeshi doctors, reflecting a pervasive stigma surrounding mental health within the medical community. This finding is consistent with a large body of studies showing that this type of stigma and the need to hide any signs of strain or burnout are ubiquitous in among medical professionals across countries and cultures (Cohen et al., 2016; Lien et al., 2020). Our findings are in line with evidence that doctors may be silent about their burnout and stress as they do not feel it is psychologically safe to have such discussions (Ng et al., 2024; Petrie et al., 2019). Our findings extend existing literature by suggesting that an absence of mental health awareness in medical education and healthcare workplace settings in Bangladesh exacerbates this problem, making it essential to integrate psychological well-being into both training curricula and workplace policies. This is crucial for LMICs especially, with a systematic review of stigma reduction interventions in LMICs showing that stigma related to mental health is reinforcing the normalisation of burnout and stress in these settings (Clay et al., 2020). This shows how cultural stigma and the subsequent workplace norms shape Bangladeshi doctors' reluctance to seek help, enforcing the narrative that burnout is a “natural” part of the profession rather than a systemic problem that requires systematic intervention—especially given the evidence of there is a widespread tendency to stigmatise and discriminate against people with mental illness in Asian countries (Lauber & Rössler, 2007).

The third theme reflected the role of high workloads and competing demands as a major source of burnout and stress among Bangladeshi doctors. These findings are in line with the Job Demands-Resources (JDR) model (Demerouti & Bakker, 2011) and the Effort-Recovery model (Meijman & Mulder, 1998) of work stress and burnout. Our findings extend existing knowledge by conducting the first exploration of this topic in Bangladeshi doctors, which suggests their job demands are excessive, while job resources are minimal or non-existent, resulting in chronic stress and burnout. A similar pattern has been reported in studies in other LMICs, which indicate that high patient loads, long working hours, and lack of institutional support drive burnout among doctors (See et al., 2018; Prabath et al., 2022; Marchand et al., 2024). For example, a study in India found that over 90% of medical professionals in a tertiary care hospital reported some level of burnout, with long hours and lack of support from seniors cited as major contributors (Grover et al., 2018). Our participants also experienced continuous high effort demands (e.g., long shifts, multiple job roles) without adequate recovery opportunities, leading to prolonged physiological and psychological strain. Similarly, a qualitative study on burnout among public-sector physicians in Sierra Leone highlighted that doctors experiencing chronic exhaustion often feel helpless due to institutional constraints, leading to detachment from their work and reinforcing a culture of silence around burnout (Jalloh et al., 2024). In this setting, overwork can become normalised, compounding stigmatised views towards reaching out for mental health support. A study in Bangladesh during the COVID-19 pandemic found that burnout, anxiety, and depression were prevalent among doctors and nurses, particularly those in high-pressure roles, suggesting that extreme work expectations and stigma around mental health are deeply ingrained in the profession (Hutchings et al., 2024). Our findings suggest that these problems are persisting after the pandemic and highlight the need for interventions that either reduce demands or increase support to tackle burnout in Bangladeshi doctors.

The last theme developed in our analysis reflects the broader factors that contribute to Bangladeshi doctors’ burnout outside of the hospital working conditions. Negative portrayals of doctors and hospitals in the media and societal misconceptions about the medical profession were found to exacerbate stress and burnout among practitioners. Participants expressed concerns over unjust blame for systemic healthcare failures, leading to a strained doctor-patient relationship. These findings support evidence from UK doctors who reported feeling demoralised by the news media's criticism of the profession (Goldacre, 2003). The fact that few studies have explored this issue in general and in LMIC contexts specifically shows that our study provides an important contribution to understanding how societal narratives influence burnout among doctors.

This finding is particularly significant as it highlights that healthcare systems do not operate in isolation - external pressures from media narratives and public discourse shape the professional and emotional experiences of healthcare workers. External pressures can help further understand why healthcare organisations suffer from poor management and dysfunctional cultures, as well as why individual interventions fail, and organisational and systemic change is needed (Montgomery & Lainidi, 2023). Further evidence from a scoping review on organisational challenges in LMICs highlighted that healthcare workers often operate in environments where societal mistrust and lack of institutional support exacerbate burnout; thus, when organisational dysfunction is combined with external scrutiny, the emotional exhaustion and stress will increase, particularly among frontline workers (Reddy et al., 2022).

### Implications

Overall, our findings clearly show that Bangladeshi doctors experience burnout in isolation and that systemic problems are at the core of their burnout and stress experiences. These findings are particularly critical as understanding burnout in low-and-middle-income countries such as Bangladesh is vital, given the disproportionate research focus on high-income countries and the unique structural challenges faced by healthcare professionals in resource-limited settings. Systemic and organisation-level interventions with a focus on organisational and policy changes in the Bangladeshi healthcare system are crucial. Empirical evidence shows that even small changes such as job and task modifications can have a positive effect on healthcare worker wellbeing and burnout (Aust et al., 2024). Moreover, the current lack of mental health awareness, combined with the negative portrayal in the media, further dehumanises Bangladeshi doctors’ professional identity; this means that action should be taken on the broader societal level, including public awareness campaigns to reduce stigma, policy reforms to safeguard doctors from unfair blame, and initiatives like educating medical journalists to foster a more balanced narrative about the medical profession in the media.

A scoping review of retention strategies for doctors in LMICs found that burnout and excessive workloads are major drivers of attrition, with financial and professional support being key to improving doctor wellbeing and retention in these settings (Jinah et al., 2024). Implementing shift-based coverage, regulating the number of workdays per month, and promoting work-life balance significantly reduced burnout levels (See et al., 2018), whereas recognition programmes, mentorship structures, and appreciation mechanisms (e.g., financial incentives and career development opportunities) have been shown to enhance morale and job satisfaction (Jalloh et al., 2024), which are critical in combating burnout In terms of raising mental health awareness, a systematic review of interventions in LMICs recommends that long-term mental health awareness programmes should be integrated into workplace policies and across all levels of education (Javed et al., 2021). Anti-stigma programmes like the “Beyond the Label” in Singapore (Subramaniam et al., 2024) have shown promising results in changing attitudes when implemented at scale and can also counteract negative media portrayals. While some interventions, such as workload management and peer support structures, have been tested in LMICs, more research is needed to evaluate their long-term effectiveness in Bangladesh specifically.

### Strengths and limitations

A key strength of this study is the diverse sample, which included doctors working in both government and private hospitals, as well as those engaged in private practice. Additionally, by including both junior and senior doctors, the study explored both early-career challenges and long-term professional struggles. However, with most respondents being based in Dhaka, the findings may still be skewed towards the experiences of urban-based doctors, who may have different access to resources, peer support, and work conditions compared to those in rural areas. Across the dataset, participants’ accounts were largely homogeneous, and this relative lack of divergent viewpoints may itself be an important finding, suggesting the consistency of structural pressures across contexts. This could also be a limitation, indicating that further research is needed to explore contrasting experiences across Bangladeshi doctors. For example, with self-selection bias being a key limitation, those who experience severe burnout but choose not to engage in discussions about their mental health might be underrepresented in the study. An additional strength of our study is the reflexive stance we adopted throughout the analytic process. In line with reflexive thematic analysis (Braun & Clarke, 2019, 2021), we view researcher reflexivity not as a limitation to be minimised but as an analytic resource that shaped interpretation and meaning making, drawing on both insider and outsider perspectives to enrich the analysis.

This study’s findings should be interpreted in light of its contextual and methodological boundaries. No transcripts or findings were returned to participants for validation, nor were any interviews triangulated with other sources. This means that some perspectives may not have been fully captured or challenged through participant feedback. It’s important to note that this decision was made deliberately to minimise the burden on doctors who were already overworked and describing experiences of stress and burnout. In addition, the multilingual nature of the study meant that transcripts conducted in Bangla required translation into English for analysis; returning translated materials for member-checking would have necessitated additional back-translation, introducing further complexity and potential bias. This approach is consistent with recent literature emphasising that member-checking should not be treated as a procedural requirement, but instead weighed carefully in light of study context (Lloyd et al., 2024; McKim, 2023).

While qualitative research allowed for the exploration of doctors’ experiences, future studies should build upon these findings by integrating qualitative research with quantitative data, such as workforce retention data, burnout prevalence rates, and patient safety indicators, to develop a comprehensive strategy for tackling burnout in Bangladesh’s healthcare system. Although our purposive sampling included doctors from different institutional contexts and specialities, the present analysis focused on shared experiences of burnout rather than systematically contrasting subgroups. Future research could build on our findings by explicitly examining how contextual factors such as hospital versus community practice, rural versus urban settings, or physician versus surgeon roles shape the experience of stress and burnout. This will allow to examine the processes and mechanisms through which these shared pressures are reproduced and sustained within different organisational settings. The experiences described reflect the healthcare, cultural, and policy context of Bangladesh and may not fully represent doctors working in other low- and middle-income countries or different health systems. While the inclusion of participants from various regions and specialities enhances the richness and transferability of the findings, they remain context-specific. Although rooted in the Bangladeshi context, the findings speak to broader questions about how structural and cultural forces shape doctors’ burnout, and their value lies in transferability and in providing theoretically informed insights that may resonate across diverse healthcare systems facing parallel challenges.

## Conclusion

Our study highlights the systemic, cultural, and social factors contributing to burnout among Bangladeshi doctors, emphasising the urgent need for structural reforms and mental health interventions. By identifying key pressure points, including the postgraduate training phase, a pervasive lack of mental health awareness and support, excessive workloads, and negative public and media narratives, our findings support the argument that burnout is deeply embedded in organisational and systemic issues. Ultimately, meaningful change will depend on collaborative efforts across healthcare institutions, policymakers, and society to create a more sustainable and supportive work environment for Bangladeshi doctors.

## Supplementary Material

COREQ.pdf COREQ.pdf

Supplemental Mat 1 Intervew Guide.docx Supplemental Mat 1 Intervew Guide.docx

## Data Availability

Fully anonymized data can be made available upon reasonable request.
