# Supplementary material for: Exploring the causes of work-related stress and burnout among doctors in Bangladesh: a qualitative study
Source: Int J Qual Stud Health Well-being. 2026 Jan 14;21(1):2616350. doi: 10.1080/17482631.2026.2616350 (PMC12810410; doi:10.1080/17482631.2026.2616350)
Supplement: Supplemental Mat 1 Intervew Guide.docx [file ZQHW_A_2616350_SM4693.docx]

**Causes of work-related stress and burnout and potential solutions - Interview Guide**

- Are you familiar with the concepts of work-related stress and burnout?
- How would you define work-related stress?
- How would you define burnout?
- If participant is unsure, explain that work-related stress is often understood as the experience of feeling that there are more demands than resources; burnout is a state of feeling exhausted due to work and/or feeling distanced from/disinterested in patients.

- What things do you think registered doctors tend to find stressful about working in Bangladesh?
- Probe for personal experiences – have you experienced work-related stress? Explore
- Have you experienced burnout? Explore
- Explore demands faced by registered doctors in Bangladesh
- Explore decision latitude/autonomy
- Probe emotional/psychological impacts of work-related stressors and burnout

- Do you think there is awareness of these stressful events in the Bangladesh medical community?
- Probe awareness in registered doctors
- Probe cultural attitudes towards stress and burnout
